# Editorial: Mortality saliency and mental health: how could awareness of death promote well-being?

**DOI:** 10.3389/fpsyt.2026.1905349

**Published:** 2026-07-13

**Authors:** Siyang Luo

**Affiliations:** Department of Psychology, Guangdong Provincial Key Laboratory of Social Cognitive Neuroscience and Mental Health, Guangdong Provincial Key Laboratory of Brain Function and Disease, Sun Yat-Sen University, Guangzhou, China

**Keywords:** culturomics, death anxiety, LLM, mortality salience, well-being

The inevitability of death is a fundamental aspect of human existence. In contemporary society, individuals are frequently exposed to death-related information via real-world crises, such as the COVID-19 pandemic, and global media coverage. This pervasive exposure makes it increasingly challenging to suppress the awareness of mortality, thereby intensifying existential anxiety. The articles compiled in this Research Topic offer a comprehensive examination of how death attitudes, mortality salience (MS), and existential concerns shape psychological processes, well-being, and social behavior, largely through the lens of Terror Management Theory (TMT).

This Research Topic highlights several critical pathways through which existential concerns are processed. Firstly, research by Gao et al. demonstrates that mortality salience increases self-objectification in both genders. They identify two underlying mechanisms: cultural worldview compliance and the suppression of creatureliness. While cultural norms explain baseline gender differences, creatureliness suppression – distancing oneself from biological reminders of mortality – functions as a universal existential defense.

Secondly, existential concerns significantly influence social and prosocial behaviors. Lu et al. posit that crisis-induced conformity acts as an affiliation defense, buffering against the distress triggered by mortality reminders. By conforming, individuals mitigate negative affect and mortality anxiety, enhancing positive emotional outcomes. Complementary to this, Chang et al. explore “altruism born of suffering,” finding that mortality salience promotes helping intentions, mediated by a search for meaning, with “negotiable fate” beliefs in Chinese culture acting as a key moderator that strengthens this prosocial response. Furthermore, Xie et al. examine the paradoxical nature of helping behavior during the COVID-19 pandemic, highlighting how death anxiety and death reflection act as competing mediators in shaping individual willingness to provide support.

Finally, the Research Topic addresses the broader implications of death attitudes for well-being and professional functioning. Wang et al. show that neutral acceptance of death positively correlates with a “good life experience,” while extrinsic goal pursuit may suppress well-being when death anxiety is high. In a professional context, Zdziarski et al. investigate the attitudes of medical personnel toward death, suggesting that professional experience and habituation contribute to varying existential perspectives among doctors, nurses, and paramedics. Lastly, Das et al. suggest that confronting death through art and meditation can transcend the pervasive denial of mortality, fostering deeper connectedness and life appreciation.

## Future directions: a cross-scale culturomics approach

Traditional terror management research has largely examined death anxiety through isolated variables (e.g., self-esteem, worldview defense) at a single psychological level. Moving forward, we advocate for Culturomics – a cross-level, interdisciplinary science that integrates micro (genetic/neural), meso (behavioral), and macro (societal/cultural) data into high-dimensional representational spaces (1). This systems perspective transcends reductionist variable-centric models and captures the holistic, networked nature of existential defenses.

First, future studies should employ Representational Similarity Analysis (RSA) to compare the geometric structure of mortality-related processes across levels (2). For example, does the representational geometry of “creatureliness suppression” in brain activity (micro) mirror the pattern of cultural worldview compliance across nations (macro)? RSA can directly test such cross-level homologies, bridging neural, behavioral, and societal data within a unified framework.

Second, Deep Neural Networks (DNNs) and large language models (LLMs) can extract higher-order, abstract cultural profiles from multi-modal inputs – including digital traces, policy responses, and historical archives (3). Rather than merely counting word frequencies, DNNs reveal latent co-occurrence patterns of existential concerns across different ecological contexts (e.g., tight vs. loose cultures, rice vs. wheat farming regions). This enables the construction of dynamic cultural profiles of death anxiety that evolve over time and space.

Third, Culturomics urges integration of the biological basis of existential culture. Genetic (e.g., 5-HTTLPR, OXTR) and neuroimaging (e.g., frontotemporal connectivity) data should be incorporated into multi-level models to examine gene−culture co-evolution under mortality salience. How do specific alleles modulate sensitivity to cultural mortality buffers, and how does this interact with collective threat (e.g., pandemics)?

Finally, adopting a systems theory perspective, future research should map the distributed, networked, and topological properties of existential defenses – from individual neurons to societal institutions. By shifting from univariate, single-level analyses to high-dimensional representation comparison, Culturomics promises to transform existential psychology into a truly integrative, cross-scale science.

In summary, this Research Topic advances our understanding of the psychological mechanisms that allow humans to navigate the reality of death. As we continue to integrate cultural, psychological, and computational approaches, we look forward to a deeper understanding of how existential awareness can be transformed from a source of anxiety into an impetus for personal growth and social cohesion.
